# Predicting Diabetic Nephropathy Using a Multifactorial Genetic Model

**DOI:** 10.1371/journal.pone.0018743

**Published:** 2011-04-14

**Authors:** Ilana Blech, Mark Katzenellenbogen, Alexandra Katzenellenbogen, Julio Wainstein, Ardon Rubinstein, Ilana Harman-Boehm, Joseph Cohen, Toni I. Pollin, Benjamin Glaser

**Affiliations:** 1 Endocrinology and Metabolism Service, Hadassah-Hebrew University Medical Center, Jerusalem, Israel; 2 Bioinformatics and Microarray Unit, The Mina and Everard Goodman Faculty of Life Sciences Bar-Ilan University, Tel Aviv, Israel; 3 The Central Bureau of Statistics, Jerusalem, Israel; 4 Diabetes Unit, The E. Wolfson Medical Center, Holon, Israel; 5 Metabolic Unit, Tel Aviv Sourasky Medical Center, Tel Aviv, Israel; 6 Soroka Hospital, Ben-Gurion University of the Negev, Beer-Sheva, Israel; 7 Endocrine Clinic, Macabbi Health Service, Tel Aviv, Israel; 8 Division of Endocrinology, Diabetes and Nutrition, University of Maryland School of Medicine, Baltimore, Maryland, United States of America; Karolinska Insitutet, Sweden

## Abstract

**Aims:**

The tendency to develop diabetic nephropathy is, in part, genetically determined, however this genetic risk is largely undefined. In this proof-of-concept study, we tested the hypothesis that combined analysis of multiple genetic variants can improve prediction.

**Methods:**

Based on previous reports, we selected 27 SNPs in 15 genes from metabolic pathways involved in the pathogenesis of diabetic nephropathy and genotyped them in 1274 Ashkenazi or Sephardic Jewish patients with Type 1 or Type 2 diabetes of >10 years duration. A logistic regression model was built using a backward selection algorithm and SNPs nominally associated with nephropathy in our population. The model was validated by using random “training” (75%) and “test” (25%) subgroups of the original population and by applying the model to an independent dataset of 848 Ashkenazi patients.

**Results:**

The logistic model based on 5 SNPs in 5 genes (*HSPG2*, *NOS3*, *ADIPOR2*, *AGER*, *and CCL5*) and 5 conventional variables (age, sex, ethnicity, diabetes type and duration), and allowing for all possible two-way interactions, predicted nephropathy in our initial population (C-statistic = 0.672) better than a model based on conventional variables only (C = 0.569). In the independent replication dataset, although the C-statistic of the genetic model decreased (0.576), it remained highly associated with diabetic nephropathy (χ^2^ = 17.79, p<0.0001). In the replication dataset, the model based on conventional variables only was not associated with nephropathy (χ^2^ = 3.2673, p = 0.07).

**Conclusion:**

In this proof-of-concept study, we developed and validated a genetic model in the Ashkenazi/Sephardic population predicting nephropathy more effectively than a similarly constructed non-genetic model. Further testing is required to determine if this modeling approach, using an optimally selected panel of genetic markers, can provide clinically useful prediction and if generic models can be developed for use across multiple ethnic groups or if population-specific models are required.

## Introduction

Diabetes Mellitus (DM) is a serious metabolic disorder, characterized by defects in both insulin secretion and action. The prevalence of the disease, which is becoming a major world-wide health problem, is increasing rapidly [1]. As a result of diabetes-associated metabolic dysregulation, many patients with type 1 and type 2 diabetes (T1DM and T2DM) develop multi-organ micro- and macro-vascular complications. These complications are the primary cause of kidney failure, adult-onset blindness and non-traumatic leg amputations in the western world [2]. Thus, diabetes and diabetic complications, particularly nephropathy, place an enormous burden on health care systems [3].

Although control of the abnormal metabolic state associated with both types of diabetes has a major impact on the incidence and severity of nephropathy, the propensity to develop this complication is, in part, genetically determined [4], [5]. As many as 25% of diabetic individuals will never develop clinical evidence of nephropathy regardless of metabolic control [As reviewed by 5]. Ethnicity plays an important role in the risk of developing diabetic nephropathy as evidenced by some racial and ethnic minorities, such as Pima Indians, Nauruan, Asian Indians, African-Americans and Mexican-Americans, having an unusually high burden of the disease [6], [7]. Familial clustering of nephropathy also suggests a strong genetic component to the risk of disease [8], [9], [10], [11]. Quantitative measures for traits related to diabetic nephropathy have exhibited moderate to high estimated heritability (h^2^): 0.30 to 0.44 for albumin-creatinine ratio (ACR) [12], [13], [14], [15], [16] and 0.36 to 0.75 for glomerular filtration rate (GFR) [14], [17], [18]. Furthermore, studies of identical twins suggest a genetic component in the pathogenesis of nephropathy in T2DM, and less so in T1DM [19].

Over the last 20 years multiple studies have identified linkage peaks in various regions of the genome or have demonstrated associations between genetic variants in different genes and diabetic complications, particularly nephropathy [20], [21]. Taken together, these studies clearly show that there is no single genetic factor that has a major effect on risk of diabetic complications in the population. Therefore, for risk prediction to be clinically useful, a composite model is needed that estimates the combined effect of “conventional” risk factors and genetic variants in multiple genes coding for proteins acting alone or interacting with each other [22].

The probability of identifying meaningful gene-gene interactions may be enhanced by selecting genes in well-defined metabolic or functional pathways that are thought to be important in the pathogenesis of the disease. For this reason, we selected genes associated with 4 metabolic pathways that are thought to play an important role in diabetic nephropathy. The methionine metabolic pathway was selected since, in addition to the potential direct cellular toxicity of high homocysteine (HCY) levels, nephrotoxicity can be caused through different mechanisms activated by this pathway including thrombotic effects and vascular damage [23]. The adiponectin pathway was selected since adiponectin levels vary in different diabetic complications making it and the genes that are responsible for its control, potentially important in the pathogenesis of nephropathy [24]. The renin-aldosterone pathway was selected since it is responsible for the blood pressure regulation, which in turn influences renal damage [25], [26], [27]. Finally, the AGEs (advanced glycation end products) pathway was selected since AGEs production and oxidative stress play an important role in the development of complications [28]. Cytokines such as *CCL5* (chemokine (C-C motif ligand 5), also known as *RANTES*, bind to their receptors in renal tissue and cause macrophage activation [29], [30], [31].

In this study, we selected a panel of single nucleotide polymorphisms (SNPs) from these 4 major pathways that were previously found to be associated with risk of diabetic nephropathy in multiple populations. After determining which of these SNPs approach nominal association with disease in our population, we created a statistical model that takes into consideration each variant and conventional risk factor alone and all possible two-way interactions. This model predicted nephropathy in our initial population, a finding that was replicated in an independent, ethnically similar population ascertained in Israel.

## Materials and Methods

### Patient populations

#### Ethics Statement

This protocol was approved by the Ethics Committees on Human Research in Hadassah-Hebrew University Medical Center, Wolfson Medical Center and Soroka Medical Center. Written informed consent was obtained from all participants.

#### Primary study populations

Patients with diabetes were ascertained by the Israel Diabetes Research Group between 2002 and 2004 from 15 diabetes clinics throughout Israel. Primary admission criteria were: (1) known diabetes (Type 1 or Type 2) for 10 or more years and (2) ethnic background, as defined by all 4 grandparents being either Ashkenazi or Sephardic-North African Jewish. Blood samples and clinical data from 1946 patients were collected. Of these, 534 samples were excluded for not fulfilling inclusion criteria, insufficient clinical data or for technical reasons such as insufficient or poor quality DNA. The clinical and demographic characteristic of the remaining 1412 subjects whose DNA was submitted for genotyping are shown in Table 1. Briefly, the overall prevalence of nephropathy was 38.9%. The majority of the patients had T2DM, which was somewhat more common in the group with nephropathy (91.2% and 83.4% in the nephropathy and non-nephropathy subsets respectively, p<0.001). Most subjects in both groups were of Ashkenazi origin (69.4% and 71.6% respectively, p = 0.40). The patients with nephropathy were slightly younger and thinner than those without nephropathy, although duration of diabetes was not significantly different in the 2 groups. Of these patients, 138 were subsequently excluded because of unsuccessful genotyping at one or more loci, leaving 1274 subjects whose data were used for model construction.

**Table 1:** Clinical and demographic characteristics of the subjects meeting all inclusion criteria and having DNA available for genotyping.

			Primary Population			Replication Population			Between Population p^3^
			Nephropathy^1^	No Nephropathy	p^2^	Nephropathy	No Nephropathy	p^2^	
**Demographic Characteristics**									
	Total number		556 (38.9%)	873 (61.1%)		296 (32.7%)	610 (67.3%)		0.0023^4^
	Male (%)		46.9	46.4	0.8278	52.7	44.8	0.0282	0.7019
	Age^5^		62.6±11.2	64.1±11.4	0.0147	61.7±14.9	58.3±17.9	0.0026	<0.0001
	Age at DM Diagnosis^5^		42.7±13.1	43.8±12.6	0.1304	40.1±17.7	38.7±18.9	0.2045	<0.0001
	Ethnic background								
		Ashkenazi Jews (%)	69.4	71.6	0.4038	100	100	—	—
		Non-Ashkenazi Jews (%)	30.6	28.4		-	-		
**Clinical characteristics**									
	Years of DM^5^		19.8±8.6	20.3±8.8	0.3498	21.6±9.8	19.6±8.9	0.0032	0.5923
	HbA1c (%)^5^		8.0±1.5	8.1±1.6	0.4553	8.52±1.58	8.12±1.45	0.0003	0.003
	Hypertension (%)		65.1	63.1	0.6824	67.6	41.5	<0.0001	<0.0001
	BMI (kg/m2)^5^		29.1±4.5	29.9 ±5.8	0.008	27.0±4.9	28.5±5.3	0.0607	<0.0001
	T2DM (%)		91.2	83.4	<0.0001	75.3	64.4	0.0011	<0.0001
**Complications**									
	Retinopathy (%)^6^		18.5	18.2	0.7776	58.8	24.6	<0.0001	<0.0001
	CHD (angina, CABG, PCI or MI) (%)^7^		30.0	47.5	<0.0001	37.5	27.5	0.0019	0.0007

1. Nephropathy  =  microalbinuria or proteinuria or end-stage renal disease (dialysis) due to diabetic nephropathy.

2. p value comparing Nephropathy and No-Nephropathy subsets of same population.

3. p value comparing total primary population to total Replication Population.

4. p value comparing prevalence of nephropathy in the 2 populations.

5. Age, age at DM diagnosis, years of DM, HbA1c, BMI are expressed in mean ± SD.

6. Retinopathy  =  For primary population retinopathy defined as proliferative retinopathy or macular edema; For replication population retinopathy defined as background or proliferative retinopathy or macular edema.

7. CHD  =  coronary heart disease, CABG  =  coronary artery bypass graft, PCI  =  percutaneous coronary intervention, MI  =  Myocardial infarction.

#### Validation study population

Ashkenazi patients with T1 or T2DM from the Hebrew University Genetic Resource (HUGR) collection (http://hugr.huji.ac.il/) were used as a validation dataset. Of a total of 1639 patients available, only 906 fulfilled our inclusion criteria, which included at least 10 years known duration of diabetes. The prevalence of nephropathy in this dataset was somewhat lower when compared to our initial dataset (32.7% vs 38.9%, p = 0.0023) as was the prevalence of T2DM. Age at ascertainment, age at diagnosis and BMI were slightly, albeit significantly lower in this dataset when compared to the primary population (Table 1). The apparent marked increased incidence of retinopathy in the validation population is due to the fact that in this population the definition of retinopathy included background retinopathy whereas in the initial population background retinopathy was excluded from this diagnosis. Complete clinical and genotype data required for analysis was available on 848 of these patients.

### Definition of nephropathy

For both the original and replication populations, nephropathy was defined as the presence of microalbinuria (0.03-0.3 g/gr creatinine), proteinuria (>0.3 g/gr creatinine) or dialysis in the absence of any other unrelated renal disease.

### Selection of genetic variants for analysis

The target candidate genes were selected according to metabolic pathways thought to be important in the pathogenesis of nephropathy (Table 2). A list of genetic variants within each gene was generated based on previously reported associations with nephropathy in other populations. This list was further restricted using the haplotype structure of the Caucasian population (CEU) in HapMap version 2 to avoid redundancy and to maximize coverage of each gene. Thus, for some genes, SNPs previously shown to be associated with nephropathy were excluded since they were adequately represented by other SNPs in high LD (r^2^>0.8).

**Table 2:** Genes/Pathways/SNPs studied.

Pathway	Gene	SNP	rs_number	MAF^1^	MAFCases	MAFContr.	Allelic Assoc.^2^p =	OR (95% CI)^3^	Logistic Regress.^4^ p =
**Vascular endothelial function/damage pathway**	*MTHFR*	677C/T	rs1801133	0.43	0.43	0.44	0.73	0.96 (0.83, 1.12)	0.63
		1298A/C	rs1801131	0.31	0.30	0.31	0.65	0.99 (0.84, 1.16)	0.87
	*MTR*	2756A/G	rs1805087	0.17	0.17	0.16	0.72	1.04 (0.85, 1.27)	0.69
	*CBS*	1080C/T	rs1801181	0.35	0.34	0.35	0.59	0.94 (0.79, 1.11)	0.46
		1985T/C	rs706208	0.40	0.40	0.39	0.87	0.99 (0.85, 1.14)	0.86
		C699T	rs234706	0.33	0.34	0.32	0.36	1.09 (0.93, 1.29)	0.28
		844ins68	rs72058776	0.05	0.05	0.05	0.79	1.12 (0.78, 1.60)	0.54
	***HSPG2*** 5	**HSPG2 A/C**	**rs3767140**	**0.16**	**0.18**	**0.14**	**0.0066**	**1.31 (1.07, 1.61)**	**0.0085**
	***NOS3*** 5	**1917G/T**	**rs1799983**	**0.23**	**0.21**	**0.24**	**0.0289**	**0.84 (0.70, 1.00)**	**0.0541**
**Adiponectin pathway**	*PPARG*	Pro12Ala	rs1801282	0.05	0.04	0.05	0.32	0.82 (0.56, 1.20)	0.30
	*ADIPOQ*	+45 T/G	rs2241766	0.20	0.21	0.19	0.20	1.17 (0.97, 1.41)	0.10
		+276 G/T	rs1501299	0.31	0.32	0.31	0.59	1.02 (0.87, 1.20)	0.77
		+712 G/A	rs3774261	0.48	0.50	0.48	0.43	1.09 (0.92, 1.30)	0.33
		-11391G/A	rs17300539	0.11	0.12	0.11	0.36	1.09 (0.85, 1.38)	0.50
		-11377 G/C	rs266729	0.25	0.25	0.25	0.96	1.02 (0.86, 1.21)	0.84
	*ADIPOR1*	-102 T/G	rs2275737	0.47	0.46	0.48	0.44	1.07 (0.92, 1.25)	0.39
		+5,843 A/G	rs1342387	0.47	0.46	0.47	0.42	0.94 (0.81, 1.10)	0.47
	***ADIPOR2*** 5	**+219 A/T**	**rs11061971**	**0.48**	**0.51**	**0.46**	**0.0176**	**1.21 (1.04, 1.41)**	**0.0135**
		+33,447C/T	rs1044471	0.47	0.45	0.48	0.10	0.88 (0.76, 1.03)	0.10
**Renin pathway**	*AGT*	M235T	rs699	0.43	0.44	0.43	0.51	0.96 (0.82, 1.12)	0.57
	*ACE*	I/D	rs4304	0.36	0.36	0.35	0.55	0.96 (0.82, 1.13)	0.61
	*AGTR1*	A116C	rs1064536	0.30	0.29	0.31	0.33	0.92 (0.78, 1.09)	0.32
**AGER pathway**	***AGER*** 5	1704G/T	Y18060	0.23	0.22	0.23	0.29	0.91 (0.75, 1.09)	0.30
		**G82S**	**rs2070600**	**0.01**	**0.01**	**0.01**	**0.68**	**1.18 (0.62, 2.69)**	**0.69**
		**2184A/G**	**rs3134940**	**0.13**	**0.15**	**0.11**	**0.0049**	**1.35 (1.08, 1.69)**	**0.0079**
	***CCL5*** 5	-28C/G	rs2280788	0.01	0.02	0.01	0.0645	1.93 (0.99, 3.77)	0.0531
	*CCR5*	-59029G/A	rs1799987	0.47	0.45	0.48	0.16	1.11 (0.95, 1.30)	0.18

1. MAF  =  Minor allele frequency determined in this dataset.

2. p values for unadjusted association with nephropathy.

3. Odds ratios are given for the comparison between the rare and common alleles. CI denotes confidence interval.

4. p value for logistic regression analysis adjusting for age, sex, duration of diabetes and type of diabetes.

5. SNPs included in the model are shown in bold.

### Genotyping

Twenty-seven variants in 15 different genes in the original dataset were individually genotyped using either PCR-RFLP or ABI Taqman™ assays. The genotyping of the validation dataset was carried out using the KASPar technology (a competitive allele specific PCR-based assay) by KBioscience (http://www.kbioscience.co.uk). Hardy-Weinberg equilibrium was evaluated using a standard one degree of freedom, two-tail χ^2^ test. The genotype successful call rate for the whole replication set (cases and controls) was 98.6% and no deviation from Hardy–Weinberg equilibrium was observed (at p = 0.05). The concordance between Taqman and KASPar-based genotyping was previously shown to be >99.5% with an error rate of <0.3%.

### Statistical analysis and modeling

#### Between group comparisons

Continuous variables were compared using the two-tailed t-test and are reported as average±SD. Discrete variables were compared using the two-tailed Fisher Exact Test.

#### Individual genotype association

After demonstrating that all SNPs were in Hardy-Weinberg equilibrium, each of the 27 SNPs was tested for association with diabetic nephropathy in an additive model by multivariable logistic regression analysis adjusting for age, sex, duration of diabetes and type of diabetes (Table 2). Five variants that approached nominally significant association with nephropathy in the primary dataset (uncorrected p value <0.055) were genotyped in the validation dataset. Analysis for association with nephropathy in the validation dataset was performed as for the primary dataset.

#### Modeling

**Figure 1:**
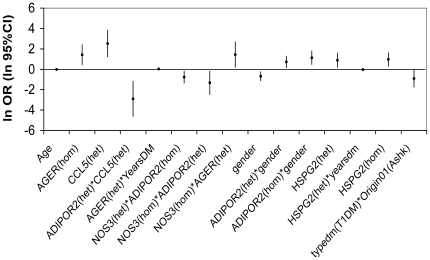
The multifactorial model: ORs and 95% CI for different SNPs and interactions in the model (expressed in logarithmic form). For the exact values see estimates in Table 3. All variables, single or interactions, contribute to the model significantly, but in different ways.

Before performing the logistic regression modeling, we recoded the genotype results to avoid loss of information for either heterozygotes or minor allele homozygotes and at the same time to distinguish between them. For each SNP we split the genotype result into two separate variables depending on the genotype result, the first defined as equal to 1 if the result is heterozygote and equal to 0 in all other cases (“het” in Fig. 1 and Table 3), and the second defined as equal 1 if the result is homozygous for minor allele and equal 0 in any other cases (“hom” in Fig. 1 and Table 3). The probability of nephropathy was calculated using the equation:
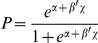



Where P is the probability of nephropathy, α is the intercept parameter, β is the vector of regression parameters and X is a matrix of the data.

**Table 3:** Model parameters with and without genetic factors.

Parameter^1^	Analysis of Maximum Likelihood Estimates			
	Estimate^2^	Standard Error^3^	Wald Chi-Square^4^	Pr > ChiSq^5^
**“Full” model**				
Intercept^6^	0.4925	0.4222	1.3608	0.2434
*ADIPOR2*(het)	0.1353	0.2059	0.4317	0.5112
*ADIPOR2*(hom)	0.1572	0.2742	0.3288	0.5663
Age	-0.0137	0.00556	6.0234	0.0141
*AGER*(het)	-0.4843	0.3674	1.7379	0.1874
*AGER*(hom)	1.4179	0.5109	7.7038	0.0055
*CCL5*(het)	2.5223	0.6635	14.4493	0.0001
*ADIPOR2*(het) * *CCL5*(het)	-2.9026	0.8759	10.9815	0.0009
YearsDM	-0.00591	0.00980	0.3638	0.5464
*AGER*(het) * YearsDM	0.0332	0.0166	3.9693	0.0463
*NOS3*(het)	-0.0251	0.1486	0.0286	0.8656
*NOS3*(het) * *ADIPOR2*(hom)	-0.7647	0.3027	6.3811	0.0115
*NOS3*(hom)	-0.3308	0.4004	0.6826	0.4087
*NOS3*(hom) * *ADIPOR2*(het)	-1.3251	0.5876	5.0866	0.0241
*NOS3*(hom) * *AGER*(het)	1.4467	0.6326	5.2296	0.0222
Gender (fem)	-0.6859	0.2393	8.2160	0.0042
*ADIPOR2*(het) *gender(fem)	0.7274	0.2947	6.0912	0.0136
*ADIPOR2*(hom) *gender(fem)	1.1368	0.3483	10.6493	0.0011
*HSPG2*(het)	0.8907	0.3650	5.9547	0.0147
*HSPG2*(het) * yearsdm	-0.0383	0.0179	4.5697	0.0325
*HSPG2*(hom)	0.9661	0.3443	7.8708	0.0050
Typedm(T1DM)	-0.1090	0.3517	0.0960	0.7567
Origin01(Ashk)	-0.0615	0.1422	0.1870	0.6655
Typedm(T1DM) * Origin01(Ashk)	-0.9075	0.4329	4.3960	0.0360
**“Conventional” model**				
Intercept^6^	0.2865	0.3116	0.8457	0.3578
Age	-0.0103	0.00486	4.5265	0.0334
Typedm1	-0.7771	0.1864	17.373	<.0001

1– The intercept and the predictor variables in the model. – see *Statistical Analysis and Modeling* section for description of how the variables were coded.

2– Binary logit regression estimates for the parameters in the model. In the logistic regression equation log[p/(1-p)] =  *a+βx* where p is the probability that nephropathy  =  1, the estimate of each variable contributes to β.

3– Standard errors of the individual regression coefficients.

4– Test statistic; the squared ratio of the Estimate to the SE of the respective predictor.

5- The probability that a particular Chi-Square test statistic (1 df) is as extreme as, or more so, than what has been observed under the null hypothesis; the null hypothesis is that all of the regression coefficients in the model are equal to zero. The numbers in the column are the associated p-values.

6– The logistic regression estimate when all variables in the model are evaluated at zero. In the above equation intercept contributes to the *α*-coefficient.

The model included 14 variables, the 5 SNPs recoded as described above (9 variables, since rs2280788 has MAF  = 0.01 to 0.02 and thus no minor allele homozygotes) and 5 independent “conventional” nephropathy predictors: diabetes type, sex of the patient, age, duration of diabetes and ethnicity. The model also allowed all possible interactions of the second degree. The best logistic regression model was chosen by the backward selection method. The final model included variables and interactions that were significant on the Wald Chi-square test as well as all variables included in the interaction terms even if these did not reach statistical significance on their own [32]. Receiver Operating Characteristic (ROC) curves were generated. To determine the impact of the genetic information on the final model, the same procedure was repeated including only the five independent “conventional” variables, allowing for all possible interactions between them. All statistical analysis was done using SAS version 9.1.

### Model validation

The model was validated internally and externally. First, the primary population was randomly divided into two groups, consisting of 75% and 25% of the study population. The larger group was used as a “training set” and the resulting model was validated on the smaller “test set”. Next, the model generated in the primary population was applied to the independent validation population of similar ethnic and environmental background.

## Results

### Association of individual variants with nephropathy

For 5 of the 27 SNPs, each representing one gene, nominal p-values obtained for association between nephropathy warranted inclusion in the model (Table 2). Although there were some differences in allele frequencies among the different ethnic origins (Ashkenazi, Sephardic or mixed); there are no differences in complication incidence among these groups, and the p values for the 5 SNPs remained essentially unchanged after adjusting for ethnicity. Thus, these 5 SNPs were used for constructing the model and for replication studies.

### Model

The best-fit model retained 9 two-way interactions, 2 of the 4 “conventional” variables (sex and age) and 3 of the 5 SNPs as significant independent variables. In addition, 9 independent variables that were included in the interaction terms but were not independently significant were included in the model (Table 3). The probability of nephropathy for each patient could be calculated using the equation:



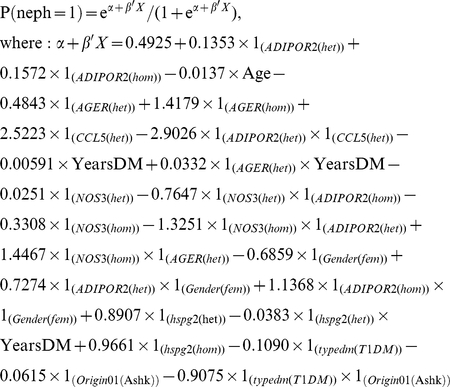



**Figure 2:**
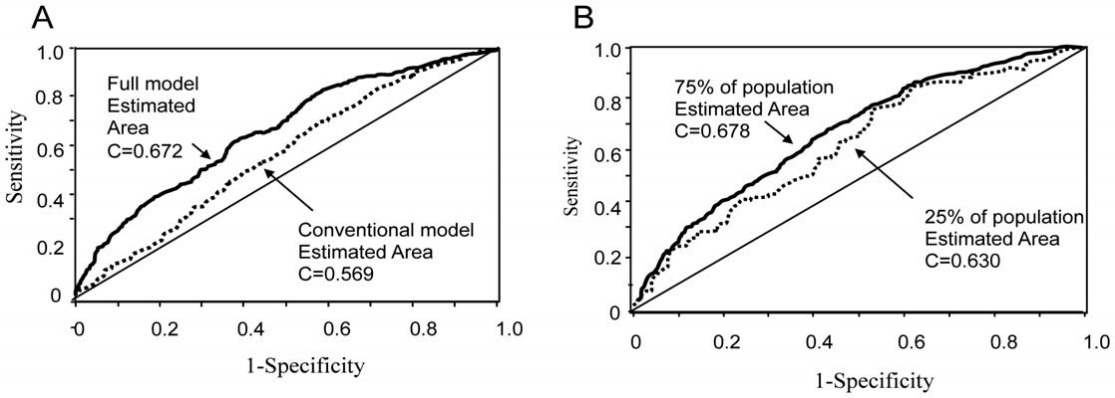
Receiver Operating Characteristic (ROC) curves in the original population. A. Predictive ability of the full and “conventional” models in the original population. ROC Curve and area under the curve (C Statistic) for “full” model (solid line; C = 0.672) and for the “conventional” model (dotted line; C = 0.569). B. Validation of the model on original population. The ROC Curve and area under the curve (C Statistic) for the model built on 75% of the original population (solid line; C = 0.678) and applied to the remaining 25% of the population (dotted line; C = 0.630). The diagonal line indicates zero predictive value of model.

The individual contribution of each of the significant single or interaction terms is shown in Figure 1. The C statistic was 0.672, indicating this model has reasonably good predictive ability (Figure. 2A).

To determine the impact of the genetic data on the model, we repeated the analysis using only the “conventional” variables (age, duration of diabetes, diabetes type, sex and ethnicity). In this case, the best model preserved only 2 conventional variables (age and diabetes type) and no interactions (Table 3). For this model, the C statistic was considerably lower (C = 0.569) indicating that the genetic data improved prediction over the conventional model (Figure 2A).

### Model Validation

The primary population of 1274 individuals was divided randomly into 2 unequal groups. The same model was rebuilt on the larger group consisting of 75% of the population (training set). The model showed a similar predictive ability when compared to the original one (C = 0.678) (Figure 2B). The ORs estimates of each variable in the rebuilt model were similar to and in the same direction as those in the original model. The model was then tested on the remaining 25% of the population and demonstrated similar predictive ability (C = 0.630) (Figure 2B).

**Figure 3:**
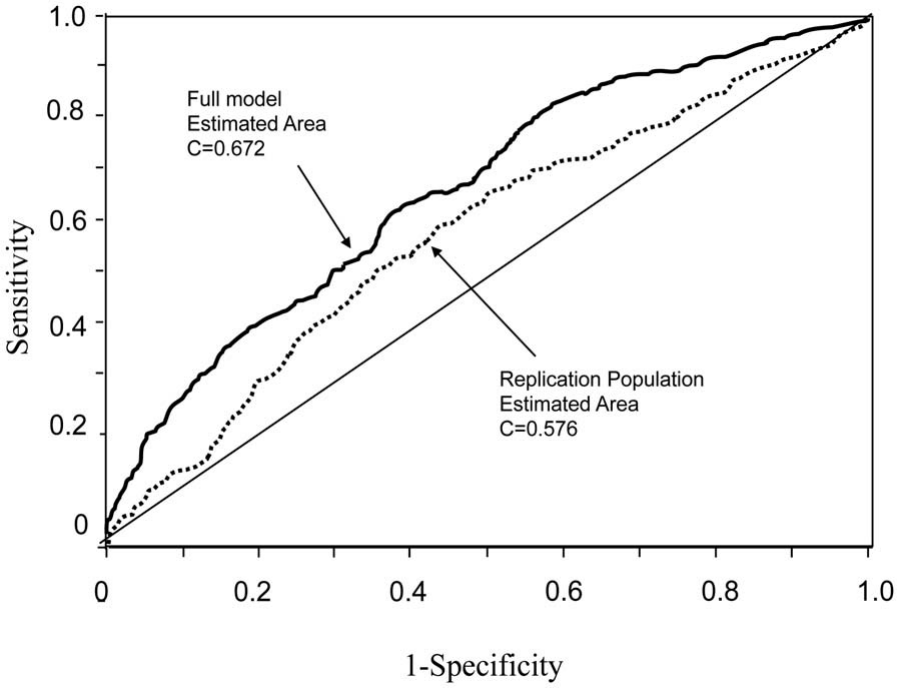
ROC Curve and area under the curve (C Statistic) for the “full” model in the replication dataset (dotted line; C = 0.576). The ROC curve and C statistic for the same model in the original population (see Figure 1A) is shown for comparison (solid line).

**Figure 4:**
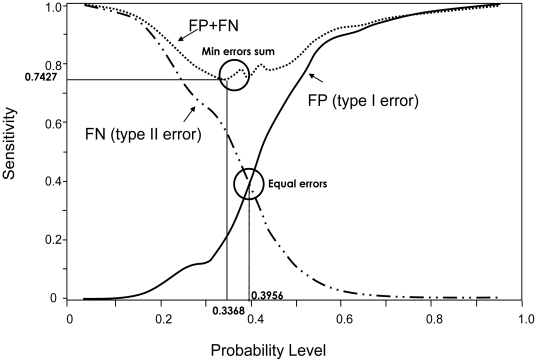
Graph of type I vs type II error. The solid line indicates the false positive rate (FP, error type I), the dashed line the false negative rate (FN, error type II) and the dotted line represents the sum of false positive and false negative rates at each probability level. The minimal errors sum is 0.7427 with probability of 0.3368.

A second validation experiment was performed on an independent population, also ascertained in Israel, but from a more restricted ethnic background (Ashkenazi Jews only). Although the ROC curve in the replication independent dataset was somewhat lower than that in the original dataset (Figure 3), we further evaluated the strength of our model, by testing it for association with nephropathy at two probability cut-offs; one corresponding to the minimal total type I and type II errors and the other corresponding to equal errors of both types (Figure 4). The model, which contains both the genetic and the conventional predictive variables, was associated with nephropathy in this population when the minimum error cut-off was used (χ^2^ = 17.79, p<0.0001), whereas the “conventional model” was not (χ^2^ = 3.27, p<0.071; Table 4). The association of the model with nephropathy using the equal error cut-off gave similar results (data not shown).

**Table 4:** Model prediction based on minimum Alpha+Beta error.

			min(Alpha + beta) (prob. = 0.3368)
**Full model including SNPs**	**Original population**	χ2	90.74
		p-value	<0.0001
		Sensitivity	82.96%
		Specificity	42.77%
		Kappa	0.2265
	**75% training subset**	χ2	63.56
		p-value	<0.0001
		Sensitivity	85.20%
		Specificity	38.54%
		Kappa	0.2136
	**25% test subset**	χ2	14.69
		p-value	0.0001
		Sensitivity	82.18%
		Specificity	39.45%
		Kappa	0.1660
	**Replication population**	χ2	17.79
		p-value	<0.0001
		Sensitivity	64.26%
		Specificity	51.14%
		Kappa	0.1319
			**min(Alpha + beta) (prob. = 0.3957)**
**Conventional model without SNPs**	**Original population**	χ2	16.9304
		p-value	<0.0001
		Sensitivity	64.55%
		Specificity	46.54%
		Kappa	0.1018
	**Replication population**	χ2	3.2673
		p-value	0.0707
		Sensitivity	36.49%
		Specificity	69.51%
		Kappa	0.0601

## Discussion

In this proof-of-concept study, we demonstrate that incorporation of multiple genetic variants, conventional risk factors and their two-way interactions into a logistic model enhances our ability to predict diabetic nephropathy.

Variants were selected based on previous publications that demonstrated significant association with diabetic nephropathy, in most cases in multiple populations of various ethnic groups. Therefore, although none of these SNPs had been tested in the Ashkenazi or Sephardic Jewish populations, the prior probability that at least some would be associated with nephropathy in our population was high. We observed significant or nearly significant association with nephropathy for 5 of the 27 SNPs tested. While none of these survived Bonferroni correction for multiple testing (alpha<0.002), the probability of at least 5/27 loci being significant at the 0.05 level by chance is 0.01.

Our finding that only 5 of these 27 SNPs replicated in our population is expected for several reasons. First, our primary population was modest in size and therefore power (e.g. less than 80% power to detect association with a SNP with an OR <1.36, minor allele frequency of 50% and alpha  = 0.05). Furthermore, although most SNP selection was based on studies in European Caucasians, of which the Jewish populations are considered sub-groups, significant genetic differences between these populations have been demonstrated [33], [34], [35]. Finally, some SNPs were selected based on studies in Japanese [36], African-American and Scandinavian (Finnish, Swedish) populations, which are likely to differ considerably from the populations studied here.

The conventional variables that were used for adjustment to estimate the main effect of the SNPs were age, sex, ethnicity, diabetes type and duration. Glycemic control was not included since HbA1c at time of ascertainment is not expected to accurately reflect overall glycemic insult to the kidneys and historical data was not available. We did not include hypertension as an independent predictor because there is a reciprocal relationship between the hypertension and nephropathy, so that hypertension increases the risk of nephropathy, while nephropathy itself can cause hypertension. Thus, in this cross-sectional study, the presence of hypertension could be the cause or the effect of nephropathy. Furthermore, the goal of genetic prediction of disease is to identify at-risk individuals before they develop co-morbidities such as hyperglycemia and hypertension.

We then tested our hypothesis that a robust predictive model can be generated by simultaneously taking into consideration multiple variables as well as possible interactions between them. Though variables that have no independent effect could interact together to produce a significant effect, we elected to use a more conservative approach and selected for our model only those SNPs that had a nominally significant or nearly significant independent impact on risk.

There are different ways by which multiple variants, genetic and “conventional”, can be combined to obtain a composite risk score. Several investigators have utilized an allele counting method in which each individual is ranked according to the number of risk alleles she/he carries in a particular set of loci, sometimes including factors that reflect the relative strength of the effect of each SNP [37], [38], [39], [40], [41], [42]. However, this method fails to take into consideration any possible interactions. In order to overcome this shortcoming, we created a logistic model that both takes into consideration the relative contribution of each factor and allows for two-way interactions. The resulting model utilized all 5 SNPs, all “conventional” variables and two-way interactions.

The model that we produced predicted nephropathy with a C-statistic of 0.672, which although not sufficiently high to be used clinically, compares favorably with published predictive models for T2DM and other complex disease [39], [40]. In sharp contrast to what was recently reported for T2DM [38], [39], our model relies heavily on the genetic component, in that removal of these factors causes the C-statistic to drop markedly. It is highly likely that a model based on a larger number of genetic variants will provide much improved prediction of disease, although theoretical studies suggest that predictive capability that will be of direct clinical utility may not be possible [43].

To determine the robustness of our model, we performed 2 independent replication analyses. First, we randomly divided our original population into two groups, a “training set” and a “test set” and showed that the model was robustly replicated in both subsets without loss of power or sensitivity (Table 4). Our second method of replication involved an independent, albeit ethnically related, population ascertained in Israel. This population differed from our initial population in that it contained only Ashkenazi Jews, as opposed to 67.7% Ashkenazi in the original population. Furthermore, a larger percentage of the replication population had T1DM and the patients with T2DM were significantly younger. Although the sensitivity and the C-statistic decreased, which is expected in a replication population that this not identical to the original population, significant association with nephropathy was still observed (χ^2^ = 17.79, p<0.0001), providing further support for the model. As expected, the “conventional” model, lacking genetic factors, was not significantly associated with nephropathy in the replication population.

Although we selected our candidate genes based on their involvement in known metabolic pathways thought to be important for the pathogenesis of nephropathy, thus hoping to enhance the probability of finding significant interactions, the rest of the modeling was performed without any intervention, with interactions selected on the basis of statistical and not physiologic criteria. Somewhat surprisingly, although our model did identify several statistically significant two-way interactions, none of these was expected based on a known physiological relationship. Interactions that were identified by the model could point to the existence of heretofore unknown functional relationships. In our model, the strongest interaction appears to involve the *ADIPOR2* and *CCL5* variants. The *CCL5* SNP natural log odds ratio estimate was 2.52 (Table 3), whereas *ADIPOR2* did not show any contribution in the model as an independent factor. However, in the framework of the model, the effect of *CCL5* SNP is entirely cancelled and even reversed by the presence of the *ADIPOR2* variant (-2.90). If this statistical interaction does reflect a physiologic relationship between these 2 genes, the mechanism is not evident. Thus, further studies are needed to determine if this interaction represents a true functional relationship and if so, how this impacts our understanding of the pathophysiology of diabetic nephropathy.

In conclusion, by studying the association between a limited panel of genetic variants and nephropathy risk, we developed a robust multifactorial logistic regression model to predict nephropathy in our study populations. This approach is unique since conventional factors were included in the model and not used only for adjustment, the impact of genetic and conventional factors was weighted according to their effect and all possible two-way interactions were allowed (genetic x genetic, genetic x conventional, conventional x conventional). Increasing the number and spectrum of variants tested would likely improve the predictive strength of the model. Use of such multifactorial models, including interactions, may pave the way to prediction of diabetic nephropathy and other complex genetic diseases in other populations. Our data in the replication population suggests that some factors in the model may be ethnicity, age or disease type dependent, indicating that the development of robust, highly predictive models may require specific adaptation of the models to different ethnic groups. They also suggest, however, that once a model is developed for a specific ethnic group, it is likely that it can be validly applied to individuals in other subsets of the same or a closely related ethnic group, further suggesting that if a highly predictive model could be developed it would be clinical useful. The ability to accurately predict the risk of nephropathy could impact the treatment approach on a patient-specific basis, thus reducing costs and increasing efficacy of individual therapeutic or preventive interventions. Furthermore, these findings may help develop a better understanding of the pathophysiology of nephropathy, thus leading to novel treatment approaches.
